# Inversion of Lake Bathymetry through Integrating Multi-Temporal Landsat and ICESat Imagery

**DOI:** 10.3390/s19132896

**Published:** 2019-06-30

**Authors:** Yuannan Long, Shixiong Yan, Changbo Jiang, Changshan Wu, Rong Tang, Shixiong Hu

**Affiliations:** 1 School of Hydraulic Engineering, Changsha University of Science & Technology, Changsha 410114, China; 2 Key Laboratory of Dongting Lake Aquatic Eco-Environmental Control and Restoration of Hunan Province, Changsha 410114, China; 3 Key Laboratory of Water-Sediment Sciences and Water Disaster Prevention of Hunan Province, Changsha 410114, China; 4 Department of Geography, University of Wisconsin-Milwaukee, Milwaukee, WI 53211, USA

**Keywords:** sounding technique, optical remote sensing, ICESat/GLAS data, water level inversion, lake bathymetry

## Abstract

Lake bathymetry provides valuable information for lake basin planning and treatment, lake watershed erosion and siltation management, water resource planning, and environmental protection. Lake bathymetry has been surveyed with sounding techniques, including single-beam and multi-beam sonar sounding, and unmanned ship sounding. Although these techniques have high accuracy, most of them require long survey cycles and entail a high degree of difficulty. On the contrary, optical remote sensing inversion methods are easy to implement, but tend to provide less accurate bathymetry measures, especially when applied to turbid waters. The present study, therefore, aims to improve the accuracy of bathymetry measurements through integrating Landsat Thematic Mapper imagery, the Ice, Cloud, and Land Elevation Satellite’s Geoscience Laser Altimeter System (ICESat/GLAS) data, and water level data measured at hydrological stations. First, the boundaries of a lake at multiple dates were derived using water extraction, initial boundary extraction, and Landsat Thematic Mapper/Enhanced Thematic Mapper (TM/ETM+) strip removal processing techniques. Second, ICESat/GLAS data were introduced to obtain additional topographic information of a lake. The striped topography of a lake’s area was then obtained through eliminating and correcting erroneous points and interpolating the values of unknown points. Third, the entire bathymetry of the lake was obtained through interpolating water level values of lake boundary points in various dates. Experiments show that accurate bathymetry (±1 m) can be successfully derived.

## 1. Introduction

Lake bathymetry provides essential information for lake basin planning and treatment, lake watershed erosion and siltation management, water resource planning, and environmental protection [1]. For measuring lake bathymetry, several sensing techniques, including underwater topographic sounding, optical remote sensing, and laser detection and ranging technology (LiDAR), have been employed. Although it has high measurement accuracy [1,2,3], underwater topographic sounding is costly and requires long survey time as professional equipment (usually a sonar sounding ship) is needed to detect lake bathymetry. As an alternative, optical remote sensing techniques can measure lake bathymetry through establishing the relationship between water depth and spectral radiations of various bands [4,5]. Despite having advantages of low cost and easy implementation, the quality of optical remote sensing imagery is susceptible to observation conditions. In addition, the inversion of topography for inland lakes is difficult to obtain due to poor water quality and high sediment content. Further, laser detection and ranging technology (LiDAR) has been employed to measure the topographical and geographical features by projecting a pulse or laser onto the sea floor [6,7,8]. However, water quality and sediment content may affect the measurement accuracy, and the application of LiDAR to inland lake areas with scarce underwater topographic resources is limited.

With the aforementioned methods, it is difficult to quickly obtain high-accuracy bathymetry information of inland lakes with complex water quality at a comparatively low cost. To address this problem, a group of innovative methods was developed to rapidly obtain the bathymetry of a lake using the periodical exposures of the lake bottom caused by changes of water levels in a lake area [9,10,11,12,13]. The water depth measurement of the lake can be directly or indirectly measured by optical remote sensing based on lake boundaries, ICESat/GLAS data, and water level data measured of the hydrological station. Remote sensing imagery was employed to extract the water boundary when the lake water level varies, and the land/water boundary can be employed to create contours of the bathymetry of a lake for topographical inversion. The method uses a single water level value to represent the water level of the entire lake on the day of remote sensing data collection. Therefore, this method is only applicable to lakes with small water level differences between upstream and downstream lake areas. When applying this method to Poyang Lake, Feng et al. (2011) developed a water level inversion method at the lake boundary for large lake areas (hereinafter referred as the Feng method) through establishing the correlation between low to medium water levels and latitude and applying the measured values from hydrological stations in the lake area to perform low to medium water level inversion of the lake area [13]. With high water, the water level in the lake area was assumed to be equivalent to the average measured water level of each station in the lake area [14,15,16,17]. So far, the Feng method is only applied to the Poyang Lake area, and whether it can be applied to other lake areas still remains to be determined. Meanwhile, the accuracy of water level inversion needs further improvements. Based on the coarse resolution Moderate Resolution Imaging Spectroradiometer (MODIS) imagery, the Feng method can only obtain relatively vague images of the details of the lake floor. To address this problem, in the present paper, Landsat imagery with higher spatial resolution is employed to obtain the boundary of a lake. Processed Ice, Cloud, and Land Elevation Satellite’s Geoscience Laser Altimeter System (ICESat/GLAS) data and measured water levels of the stations were employed as the water level control point for the day of remote sensing imagery acquisition. Multi-day water levels of lake boundary points were obtained using different water level inversion methods (the Feng and kriging interpolation methods). Finally, the rapid acquisition of the lake bathymetry was achieved by the interpolation of a large number of boundary points with elevation values. Kriging interpolation uses a variogram to spatially interpolate variables from a geostatistical perspective and is widely used in various fields [18,19,20,21]. The introduction of kriging interpolation made it possible to establish the relationship between water levels at the control points and across an entire lake. If a suitable variogram can be found based on water levels at control points, kriging interpolation can be employed to invert the water level at each point on the lake boundary, and subsequently improve the accuracy of the bathymetry of a large lake.

The next section of this paper introduces the study area and data acquisition techniques. Then, the basic principles and experimental methods—including Landsat image processing, waterbody extraction, Landsat 7 ETM+ imagery strip processing, ICESat/GLAS data error point elimination, and projection conversion through correlation analysis—are described. Next, the acquisition of corresponding water level control points, inversion of the lake water level, superposition of multi-day water level boundary points, and interpolation methods were applied to obtain the bathymetry of the lake. Results and accuracy assessment and comparative analysis are detailed in the next section. Finally, a discussion and conclusions are presented.

## 2. Study Area and Data

**Figure 1:**
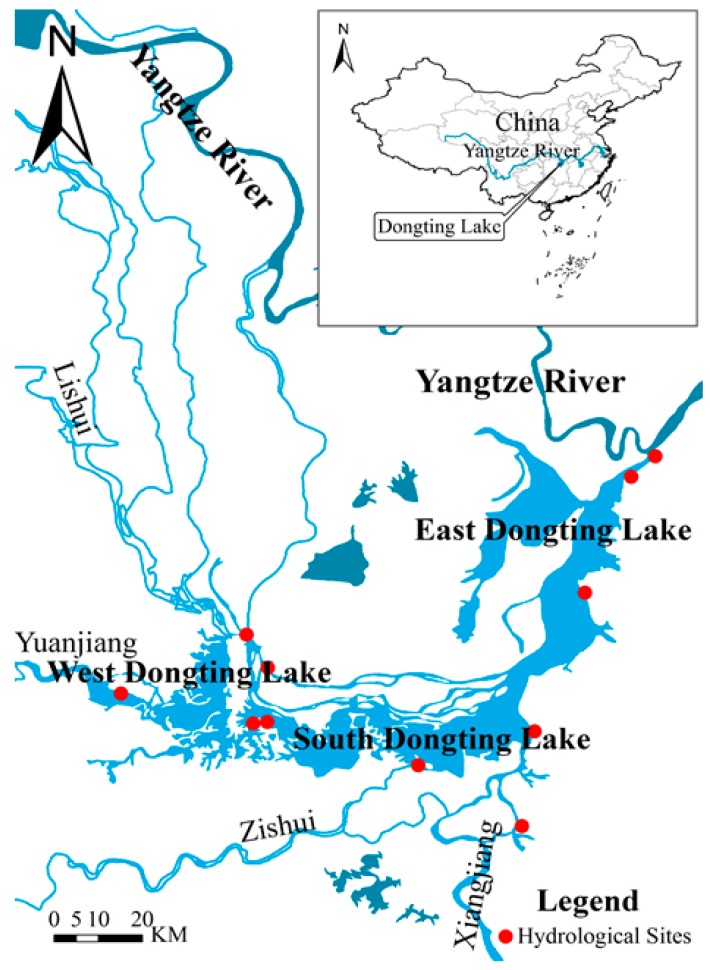
Study area and distribution of hydrological stations.

Dongting Lake lies on the south bank of the middle reaches of the Yangtze River and spans Hunan and Hubei provinces (27°39′–30°25′ N, 111°19′–113°34′ E), China (see Figure 1). The eastern, southern, and western parts of the lake combine to form the second largest freshwater lake in China. The Songzi, Hudu, and Ouchi rivers discharge water and sand from the north and middle reaches of the Yangtze River into the lake from the three entrances of Songzi, Taiping, and Ouchi, respectively, forming the Sankou water system. To the south and west, the Xiangjiang, Zishui, Yuanjiang, and Lishui rivers enter the lake, forming the Sishui water system. The water flows into the lake and is then discharged into the Yangtze River at the Chenglingji Lake outflow. Dongting represents a typical inflow and outflow lake [22].

Dongting Lake, with important ecological functions and economic value [23,24], is included on the List of Wetlands of International Importance by the United Nations Educational, Scientific, and Cultural Organization. Dongting Lake is one of the two remaining natural lakes contributing to the rivers of the middle and lower reaches of the Yangtze River. As such, it plays an extremely important role in regulating flood runoff in the Yangtze River system and in protecting genetic material of many species and biodiversity; this region forms one of the most concentrated areas of biodiversity in China.

Over the past few decades, floods and sediments from the SiShui and Sankou branches of the Yangtze River have caused constant topographical changes to Dongting Lake. In addition, human activities such as cascade reservoirs, sand mining, and reclamation have caused frequent changes in the bathymetry of the lake. Changes in lake area and volume have weakened ability of Dongting Lake to regulate runoff and floods [25,26]. Sedimentation and related human activities in the Dongting Lake area along with the complex topography make it necessary to estimate the topography of Dongting Lake by remote sensing methods [27,28].

Suspended sediments and the complex composition of Dongting Lake waters can affect the results of traditional remote sensing used for bathymetry inversion [29,30,31,32]. In addition, the water level in the lake area changes frequently during any given year, varying by nearly 10 m between the low- and high-flow periods. This variable water level provides an opportunity to acquire the bathymetry of the lake indirectly [33]. At the same time, the large geographical spatial extent of the Dongting Lake area means that the data of a single hydrological station cannot represent the water level of the entire lake area, making inversion of the lake water level difficult.

The data used in the present study includes remote sensing imagery, measured water level data in the lake area, and measured bathymetry of the lake. Remote sensing data include 20 Landsat TM and Landsat ETM+ images from 2001 to 2004 (Table 1), and four orbital elevation data from the ICESat/GLAS satellite covering the Dongting Lake from 2003 to 2004. Due to the complicated hydraulic conditions and the large variation of water level in Dongting Lake, especially the absence of hydrological stations in many regions (Figure 1), it is necessary to increase the number of water level control points. Considering that the track of the ICESat/GLAS satellite passes through the East Dongting Lake and the South Dongting Lake, the water level data in the region without hydrological stations can be indirectly provided (the water level data extraction method is described in detail in Section 3), thus effectively increasing the number of water level control points for the day. Although a larger number of remote sensing images and ICESat/GLAS data will provide more topography interpolation data, serious erosion and deposition phenomenon in the Dongting Lake occurred [26]. As a result, the bathymetry of the Dongting Lake changes dramatically. If the time span of remote sensing data used for inversion is too long, topographic changes due to erosion and deposition cannot be ignored. Therefore, this paper comprehensively considers the data volume and time span and selects the remote sensing image and ICESat/GLAS data for topography inversion from 2001 to 2004.

It should be noted that the SLC error in the ETM+ data (after 2003) was not corrected in this study. Although the image is partially missing, it can still be used to extract the boundary of the lake. Based on the principle of covering the boundary point data and lake area as much as possible, we still employed the Landsat ETM+ of rare-cloud period after 2003. The Dongting Lake area spans two Landsat scenes, numbered 124/39 and 124/40. Radiometric calibration and atmospheric correction were initially carried out, and the images were cropped and resampled to facilitate the later waterbody index calculation using ENVI 5.3.1 (Harris Geospatial Solutions, Inc., Boulder, CO, USA), commercial image processing software. The ICESat/GLAS satellite data was downloaded from the United States National Snow and Ice Center GLA14 L2 data products with three classes and 15 categories. The information about the ranging properties in the product was viewed by the official data viewing tool, HDF View 2.13 (The HDF Group, University of Illinois at Urbana-Champaign, Champaign, IL, USA). In addition, the measured lake water level data was acquired from 10 monitoring stations of the Dongting Lake area from 2001 to 2004, including: Chenglingji, Yueyang, Lujiao, Yingtian, Xiangyin, Caowei, Yangliutan, Yuanshui, Nanzui, and Xiaohezui. The data were converted to the Yellow Sea Elevation System for further processing.

The reference bathymetry data represents the measured 1:10,000 topographical data of the Dongting Lake area in 2003. The elevation point coordinate system was converted into the WGS 84 coordinate system using the ArcGIS 10.3 software Projections and Transformations tool (ESRI, Redlands, CA, USA). Based on the data product’s own attribute information, laser footprints with good reliability and high elevation accuracy were screened by a multi-criteria constraint algorithm.

## 3. Methods

**Figure 2:**
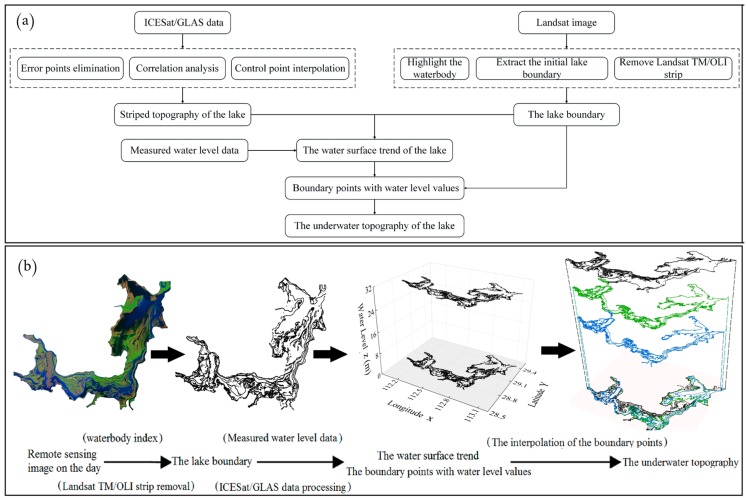
Diagram of terrain computation principle: (**a**) technical roadmap, (**b**) process diagram.

**Figure 3:**
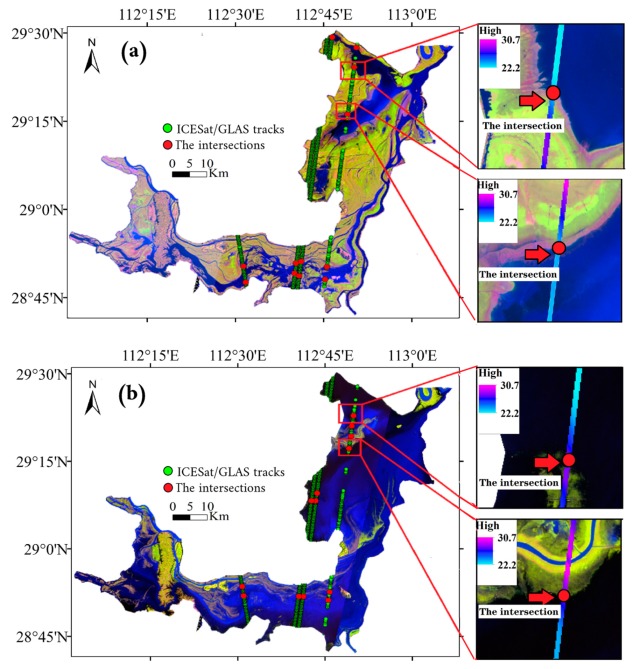
Intersections of ICESat/GLAS with lake boundaries at different water levels in the Dongting Lake area: (**a**) low water level, (**b**) high water level.

**Figure 4:**
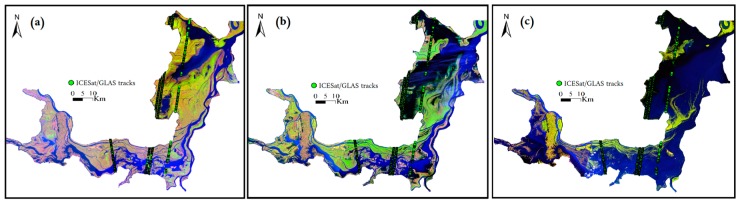
Landsat thematic mapper red–green–blue images showing three different lake inundation conditions: (**a**) low water level, (**b**) moderate water level, (**c**) high water level.

The developed method (Figure 2) can be divided into several steps. First, the Automatic Water Extraction Index (AWEI) was applied to the Landsat TM/ETM + image to extract the lake boundary for the day of collection. Second, water level data at control points, including the measured water level data of the hydrological station and the water level data obtained indirectly through ICESat/GLAS [9]. The specific steps are as follows: (1) obtaining topographic information above the lake surface (in dry season) through employing ICESat/GLAS satellite data; (2) deriving the elevation at the intersection of the topography profile and multi-period lake water boundary; (3) treating the intersection point as a virtual hydrological station (Figure 3), and measuring the elevation of the intersection point similar to the water level data monitored at hydrological stations. ICESat/GLAS has higher accuracy on land, and the topographic profile of lakes is obtained through ICESat/GLAS data. The intersection elevation data measured using the ICESat/CLAS and the hydrological station data measured at the same date were summarized to form the data set of water level control points in the lake. As shown in Figure 3, the color strip shows the elevation of the topography profile with a local magnification. It is worth noting that topography profile can intersect with the multi-period lake boundary to generate multiple water level control points corresponding to different dates (Figure 4). For example, the lake boundary extracted by the Landsat imagery intersects with ICESat/GLAS satellite data at 12 elevation points that can be used for water level inversion (Figure 3a). Similarly, as shown in Figure 3b, the lake boundary extracted from another day’s Landsat imagery has 13 intersections with ICESat/GLAS satellite data, which can also be used for water level inversion. In general, combining Landsat imagery with ICESat/GLAS data, we have obtained more than 100 elevation points for water level inversion. Next, the water level trend of the lake area is inversed using the corresponding date control point water level data to distribute the final water level of the data collection day to the lake boundary point. The sounding of the lake is then obtained by inserting a boundary point with a water level value. The full process will be covered in the following sections.

### 3.1. Lake Boundary Extraction

**Figure 5:**
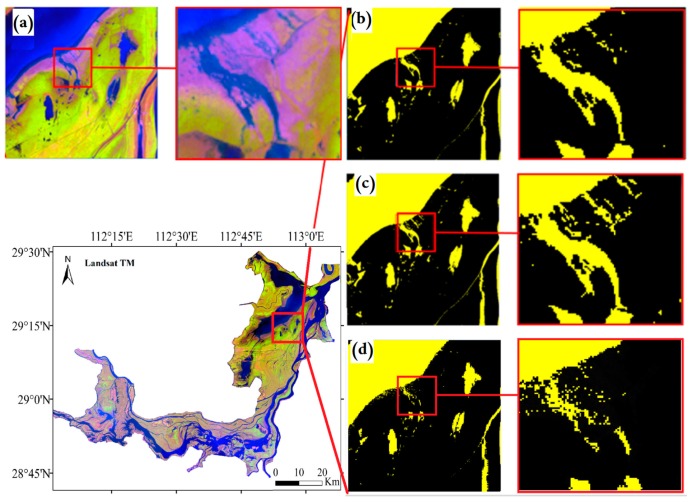
Comparison of the extraction results with (**a**) Landsat TM RGB imagery, (**b**) AWEI, (**c**) MNDWI, and (**d**) NDWI in the East Dongting Lake.

The waterbody index method was employed to extract water areas from remote sensing imagery. Many waterbody indices are currently available. In this paper, we applied three indices, Normalized Difference Water Index (NDWI), the Modified Normalized Difference Water Index (MNDWI), and Automated Water Extraction Index (AWEI), as the water masks (Figure 5). Figure 5 indicates that AWEI has a satisfactory result in extracting the water body of the Dongting Lake through visualization and accuracy analyses. The advantage of the AWEI is the distinction of water shadow and threshold selection [34]. Due to the suspended sediment and pollution, some areas of the Dongting Lake could not be accurately identified by NDWI and MNDWI, while AWEI performs better.

We chose the Automated Water Extraction Index (AWEI) [34] to extract the boundary of Dongting Lake. Studies have shown that when the AWEI is applied to the water body extraction in the Dongting Lake, the threshold value is set between −2300 and −2200. In addition, in order to ensure the clear and complete extracted boundary, images with cloud cover of less than 10% were selected in this paper.

(1) AWEInsh=4×(band2−band5)−(0.25×band4+2.75×band7)

(2) AWEIsh=band1+2.5×band2−1.5×(band4+band5)−0.25×band7

where *AWEI_nsh_* was applied to identify water pixels from non-water pixels, including buildings; and *AWEI_sh_* was further employed to remove remaining non-water objects (e.g., shadows, etc.), which are easily confused with water body.

### 3.2. ICESat/GLAS Data Processing

ICESat/GLAS data processing and land/water intersection level data acquisition mainly includes following four steps: (1) erroneous point elimination, (2) coordinate system conversion using correlation analysis, (3) ICESat/GLAS based striped topography generation, and (4) waterbody boundary intersection point acquisition. Details of these steps are described as follows.

#### 3.2.1. Erroneous Point Elimination

ICESat/GLAS satellite is equipped with the Earth Laser Altimetry System, GLAS, which serves as the only laser altimeter satellite for earth observation. In the process of transmission through the atmosphere, various factors such as the atmosphere and clouds may affect the laser’s ranging ability, therefore resulting in errors in its ranging accuracy and available elevation data. Therefore, it is necessary to screen the elevation data for better accuracy [35].

**Figure 6:**
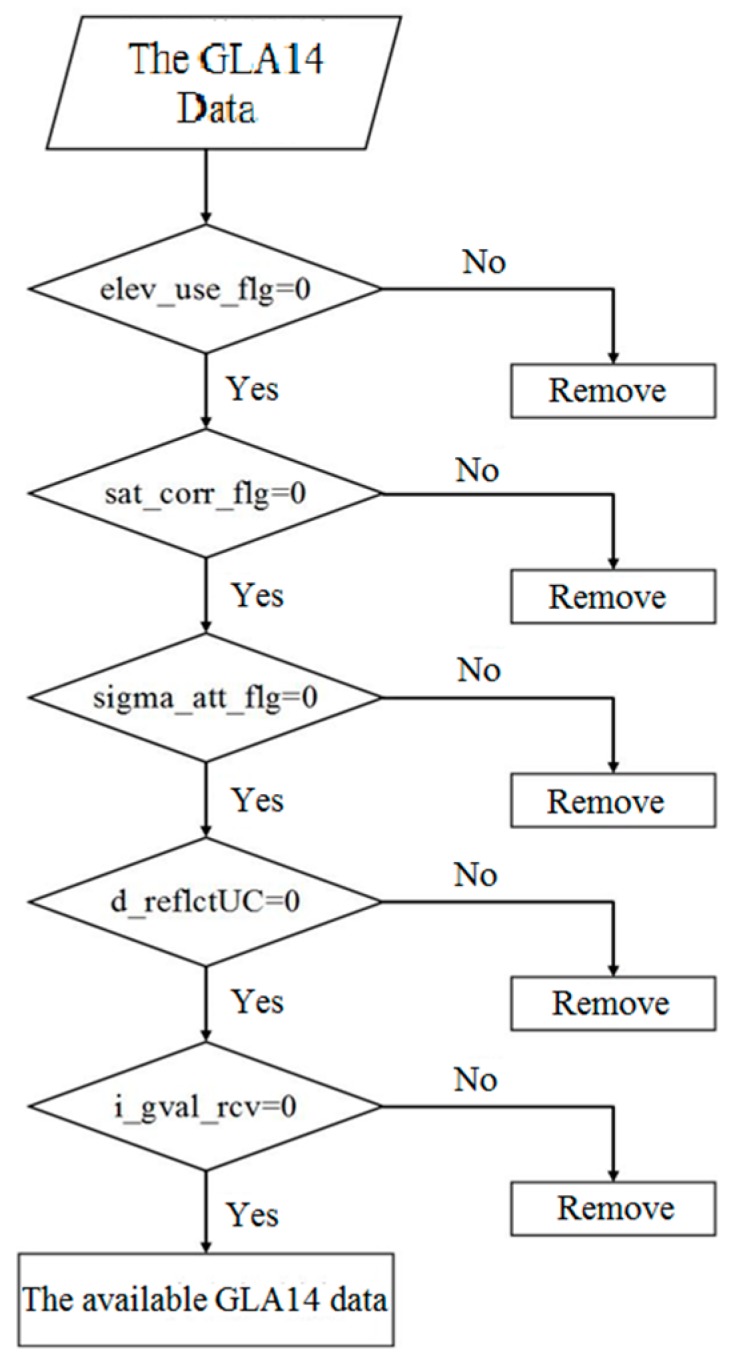
Workflow of multi-criteria constraint on Ice, Cloud, and Land Elevation Satellite’s Geoscience Laser Altimeter System data.

For improving the data accuracy, a multi-constraint algorithm has been applied to screen the ICESat/GLAS data [36]. In this paper, we employed an adjusted multi-constraint algorithm to achieve data screening. As shown in Figure 6, elevation availability (elev_use_flg), saturation correction (sat_corr_flg), attitude quality index (sigma_att_flg), ground surface reflectance (d_reflectUC), and atmospheric scattering gain (i_gval_rcv) are employed to remove the erroneous points of the ICESat/GLAS data.

#### 3.2.2. Coordinate System Conversion using Correlation Analysis

The ICESat/GLAS altimetry data are with the Ocean TOPography EXperiment (TOPEX)/Poseidon ellipsoid and the EGM 2008 elevation, while the measured topographical data are with the Yellow Sea 85 elevation. Therefore, it is necessary to unify these two coordinate systems. Song provided a correlation analysis method to convert the coordinate systems and achieved good results [37]. Therefore, in this study, we used correlation analysis to test the values of six evenly distributed ICESat/GLAS data points (Ocean TOPography EXperiment (TOPEX)/Poseidon ellipsoid) and measured elevation data (EGM 2008 elevation) at the same location. It is helpful for the unification of geodetic reference systems. Then the correlation analysis was used to correct the ICESat/GLAS altimetry data and elevation. The altimetry data under TOPEX/Poseidon ellipsoid and the Earth Gravity Model the 2008 level were converted to altimetry data under the Huanghai 85 elevation system.

#### 3.2.3. ICESat/GLAS based Striped Topography Generation

This paper selected four relatively independent orbits of the ICESat/GLAS satellite in the Dongting Lake area from 2003–2004 and obtained orbital elevation data (Figure 3). Through implementing the steps of elevation system screening and elevation coordinate system conversion, a total of 1373 elevation points were generated, and imported into ArcGIS 10.3 for visualization and interpolation. Finally, the terrain profile of the ICESat orbit was obtained using ordinary kriging interpolation which provides support for the intersection of the terrain profile and the multi-day lake boundary.

#### 3.2.4. Waterbody Boundary Intersection Point Acquisition

The above operation allowed the striped bathymetry of the lake at the ICESat/GLAS footprint to be obtained. In this paper, the extracted lake boundary on the day of data acquisition was superimposed on the striped topography. The intersection point was taken as the additional control water level point for the day of data acquisition. According to the positions of the hydrological station, the measured water level, and the lake boundary, the water level control points with coordinates and water level information were generated, and the water level control points indirectly obtained through ICESat data were combined to improve the accuracy of lake water level inversion.

### 3.3. Water Level Inversion

The accuracy of water level inversion directly affects the final results of the inversion of the bathymetry of the lake. This paper employed two water level inversion methods: the Feng method and the kriging interpolation method. Through the comparison of the water level inversion results and an analysis of the final results of topography inversion, we hoped to find a suitable water level inversion method for the lake area.

When estimating the bathymetry of Poyang Lake based on the water level and the latitude of the control hydrological stations, Feng established a function relating water levels and latitudes to carry out the boundary water level inversion. The estimation of the bathymetry of Poyang Lake proved that for large lakes, water levels and latitude have a close correlation. The kriging interpolation method, also known as the spatially local interpolation method, provides a method for unbiased optimal estimation of regionalized variables in a finite region based on the variogram theory and structural analysis. The following sub-sections introduce the above two water level inversion methods in detail.

#### 3.3.1. Feng Method

The Dongting Lake area has a significant difference in water level between the upper and lower reaches. Previous studies have shown that the three lake areas that comprise Dongting Lake have different hydrological connections. Based on the idea of Feng’s linear regression method and the characteristics of Dongting Lake, the present study investigated the correlation between water level and latitude of East Dongting Lake. We also analyzed the correlation between water level and longitude of West and South Dongting Lake, established a regression equation of water level and geographical factors, and inverted the water level of the lake boundary points based on the latitude and longitude of those points.

#### 3.3.2. Kriging Interpolation

Kriging interpolation provides a method for unbiased and optimal estimation of regionalized variables in a finite region based on variogram theory and structural analysis [38]. The kriging interpolation method includes eight kinds of interpolation methods, such as the ordinary and universal kriging interpolation methods. The ordinary kriging interpolation method has been most commonly used. In contrast, the universal kriging interpolation method is mainly used for spatial variables with drift phenomenon. That is, the mean of the variable is a spatial variable that varies with position. To determine whether the water level data were suitable for kriging interpolation and whether an obvious drift phenomenon occurred, this paper used both the ordinary and universal kriging interpolation methods to simultaneously perform water level inversion and evaluated the inversion results.

### 3.4. Acquisition of Lake Topography

From the above operations, a large number of elevation control points reflecting the bathymetry of the lake were obtained. The bathymetry of the lake was then obtained by interpolating the elevation control points. The accuracy of bathymetry inversion of the lake depended on the accuracy of the lake boundary extraction and the assigned water level of the boundary points.

## 4. Results

### 4.1. Landsat Remote Sensing Image Processing

**Figure 7:**
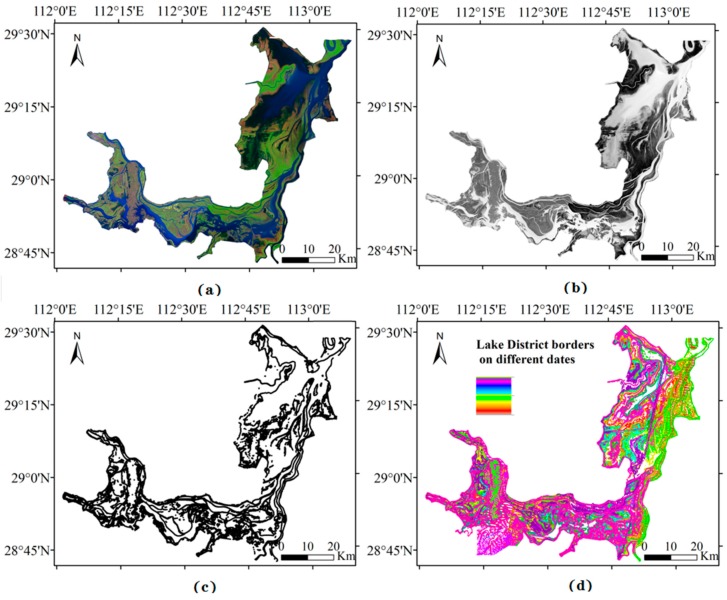
Landsat remote sensing image processing: (**a**) Landsat Thematic Mapper red–green–blue imagery; (**b**) improved waterbody index (AWEI); (**c**) Landsat ETM lake boundary extraction; (**d**) superimposed effect of the lake boundary in multi-day.

Through applying AWEI, we extracted the boundary of the Dongting Lake as shown in Figure 7a,b. It indicates that AWEI can effectively highlight the waterbody in the study area. The lake boundary (Figure 7c) extracted based on AWEI can effectively reflect the corresponding characteristics of the lake area under different water levels, which can be associated with lake bottom information. Moreover, the accuracy of bathymetry of the lake is highly associated with the number of lake boundaries with different water levels. In fact, the more the number of lake boundaries, the more comprehensive the coverage of the lake bottom, and the clearer the topography. Figure 7d indicates that the lake area boundaries cover most areas of Dongting Lake, except few deep-water areas.

### 4.2. ICESat/GLAS Data Screening

**Figure 8:**
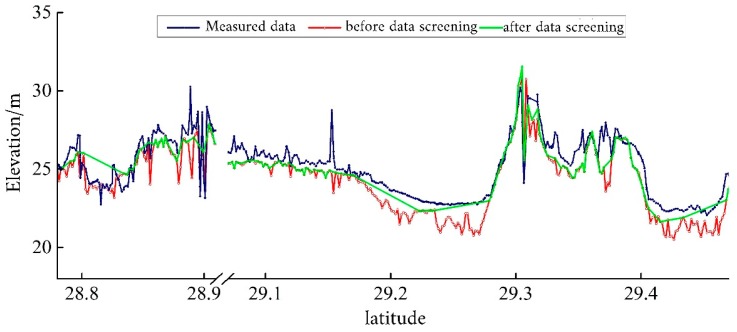
Comparison of the elevation before and after data screening of Ice, Cloud, and Land Elevation Satellite’s Geoscience Laser Altimeter System data at the footprint.

The ICESat/GLAS satellite laser is susceptible to atmospheric and cloud layer interference during the round-trip process, resulting in ranging errors. The data processing steps described in Section 3.2 were applied for data screening. A comparison between before and after screening (Figure 8) illustrates that the ICESat/GLAS data processing can effectively remove erroneous data points. In order to reflect the data screening effect in combination with the measured elevation data, the topographic lines before and after the screening were directly connected by the elevation point data without interpolation between elevation points.

**Figure 9:**
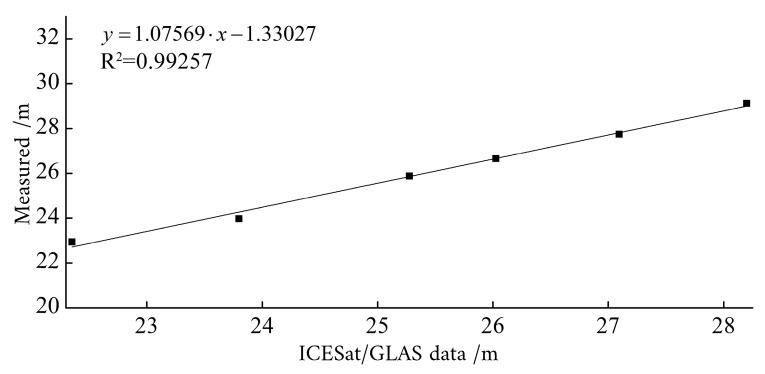
Ice, Cloud, and Land Elevation Satellite’s Geoscience Laser Altimeter System data and elevation data correlation analysis.

**Figure 10:**
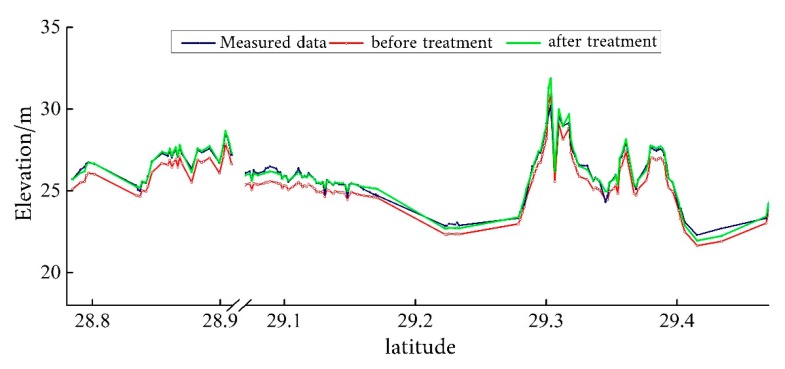
Comparison of Ice, Cloud, and Land Elevation Satellite’s Geoscience Laser Altimeter System data before and after correction as well as the measured elevation data.

After the data screening, the coordinate systems of the ICESat/GLAS data and the measured elevation data have been unified through correlation analysis. A good correlation existed between the ICESat/GLAS data and the measured elevation data (Figure 9). Through applying the regression equation, the coordinate system of the ICESat/GLAS elevation data was converted to the Yellow Sea 85 elevation coordinate system, consistent with the measured elevation data. Figure 10 provides a comparison of the ICESat/GLAS data between before and after correction, and the measured elevation data. After the elevation correction was performed, the ICESat/GLAS data was basically consistent with the measured elevation data.

### 4.3. Water Level Inversion

The accuracy of water level inversion directly affects the final bathymetry inversion of the lake. With the two water level inversion methods, the Feng method and kriging method, the results of bathymetry inversions are described as follows.

#### 4.3.1. Feng Method

**Figure 11:**
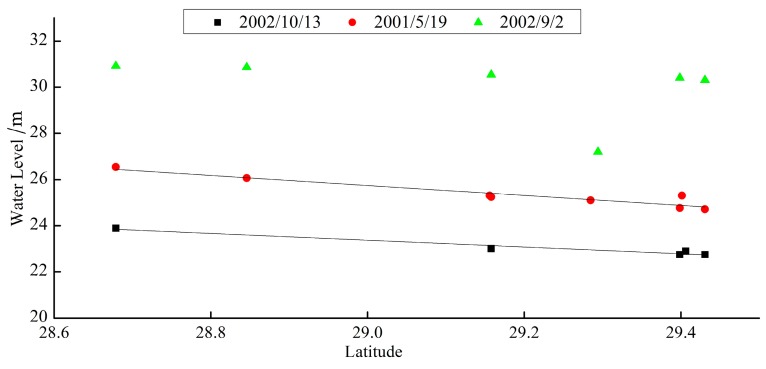
Relationship between latitude and water level in low-flow period, normal-flow period, and high-flow period.

**Figure 12:**
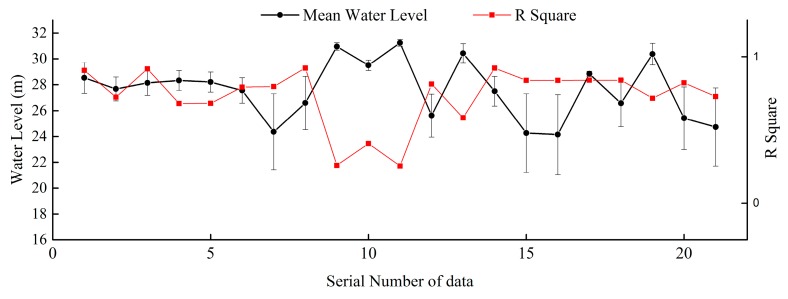
Relationship between water level and correlation coefficient during the study period.

The correlation between water level and latitude for the East Dongting Lake, as well as the correlation between water level and longitude for the west and south Dongting Lake, were investigated. A regression equation showing the relationship between water level and geographical factors was established. The water level, i.e., the elevation of the land/lake boundary point, was inverted based on the latitude and longitude of that point. Preliminary correlation analysis was carried out on the three-day water levels during the low-, normal-, and high-flow periods. Figure 11 shows that the water level in the Dongting Lake area was correlated to geographical factors. The correlation is high at low and middle water levels, but relatively low with high water levels (Figure 12).

Referring to the results shown in Figure 12 and combined with the concept of water level inversion provided by Feng, if R-squared value is less than 0.5 (high water level period, we used the average water level of the lake to represent the water level of the entire lake. When the R-squared value is greater than 0.5 (medium and low water level periods), the relationship between latitude and longitude and water level was fitted. Based on the fitting relationship as well as the latitude and longitude of the lake boundary point, the water level at the lake boundary point was inverted to obtain the lake boundary point with the water level value.

#### 4.3.2. Kriging Model

We used Trend Analysis of the Geostatistical Analyst in ArcGIS 10.3 to analyze the data, including quantile-quantile plot (QQPlot) diagram, Histogram, Voronoi diagram, and global trend analysis. The water level data was found to have a U-shaped curve trend in the north–south and east–west directions. We used the Geostatistical Wizard to perform kriging interpolation, selected the corresponding kriging method in the kriging type column, selected ‘log transformation’ in transformation, and selected second in the order of trend removal to fit the U-shaped curve trend of the water level data in the north–south and east–west directions. Next, a variogram was selected, including spherical and exponential, and the anisotropy was selected. A four-point elliptical neighborhood was used to search in interpolation. The minimum and maximum numbers of search points per neighborhood were set to two and five, respectively. Finally, the water surface trend of kriging interpolation could be obtained.

With the same variogram, the fitting effect of the universal kriging interpolation method was better than that of the ordinary kriging interpolation method (Table 2). This shows that a certain amount of drift occurred in the water level data when the number of water level sample points was small, and that the universal kriging interpolation method is more suitable for water level inversion. Simultaneously, after multiple interpolations, a higher inversion accuracy could be obtained in most cases with the application of a spherical variogram. In summary, the universal kriging interpolation can better fit the spatial variation of the water level.

### 4.4. Water Level Inversion

**Figure 13:**
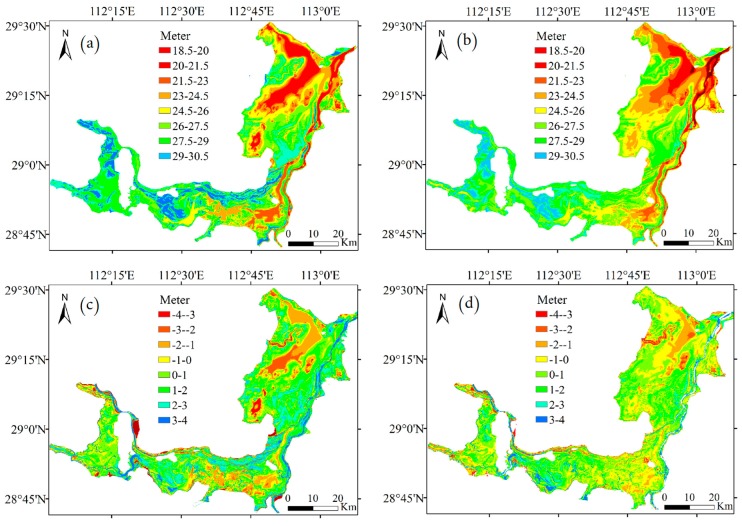
Lake bathymetry model in the Dongting Lake area: (**a**) Feng model; (**b**) kriging model; (**c**) distribution of the difference in the water level between the Feng and measured elevation grid models; (**d**) distribution of the difference in the water level between the kriging and measured elevation grid models.

Using Landsat and MODIS series remote sensing image data, the lake boundary was extracted and converted into discrete points through ENVI and ArcMap. Using trend surface analysis and kriging interpolation, the water level of each lake boundary point on the day of data acquisition was calculated by the water level of each control point and the boundary latitude and longitude. Then the discrete elevation points with latitude and longitude and water level were obtained. The discrete points were rasterized to generate the topography and elevation data (Figure 13a,b). By superimposing and subtracting the topographic raster map obtained by the two methods with the measured topographic raster map, the elevation error distribution map was obtained, which can clearly show the accuracy of topography inversion by the two methods (Figure 13c,d). The comparison shows that the topography inverted by the kriging method is more consistent with the measured topography, and with higher accuracy.

**Figure 14:**
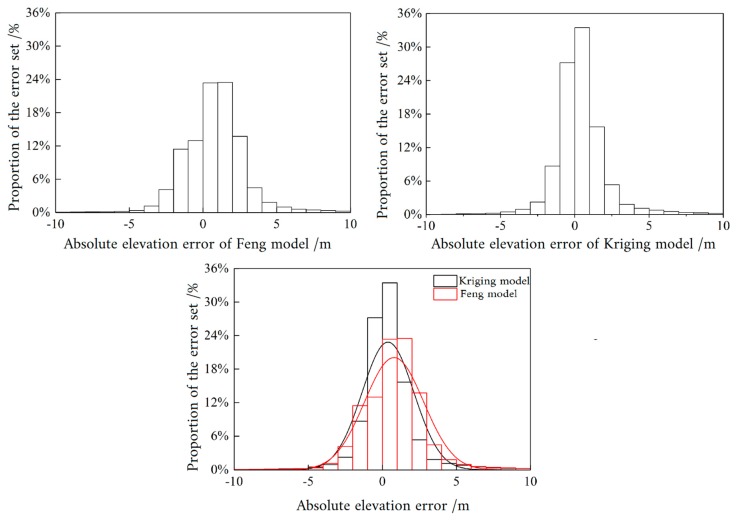
Histogram of absolute elevation error.

To compare the accuracy of the two models in more detail, a measured elevation checkpoint was used for accuracy evaluation. The kriging and the Feng models were interpolated separately to obtain the elevation at the checkpoint. Figure 14 shows the statistical histograms of the elevations of the checkpoints obtained by interpolating the two models and the measured elevations of the checkpoints. Because the bathymetry of the lake is complex and variable, the difference between the inversion results of the two models and the measured topography elevation was defined as the absolute error elevation, which was used as the evaluation index. The range of the absolute error elevation of the kriging model was small, and the distribution was concentrated mainly in the range of −1 to 1 m, accounting for about 60% of the entire error set (Figure 14). The absolute error in elevation of the Feng model was large in the concentration interval, and was mainly distributed in the range of −2 to 3 m. The kriging model was more accurate than the Feng model in the bathymetry inversion of the lake.

## 5. Discussion

### 5.1. Accuracy Evaluation of Terrain Inversion Method

**Figure 15:**
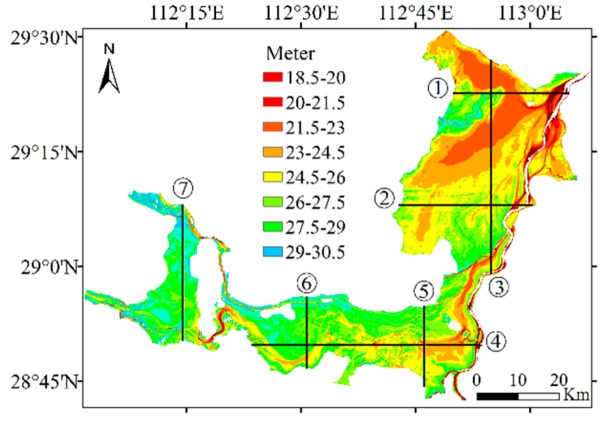
Text areas and check cross section.

**Figure 16:**
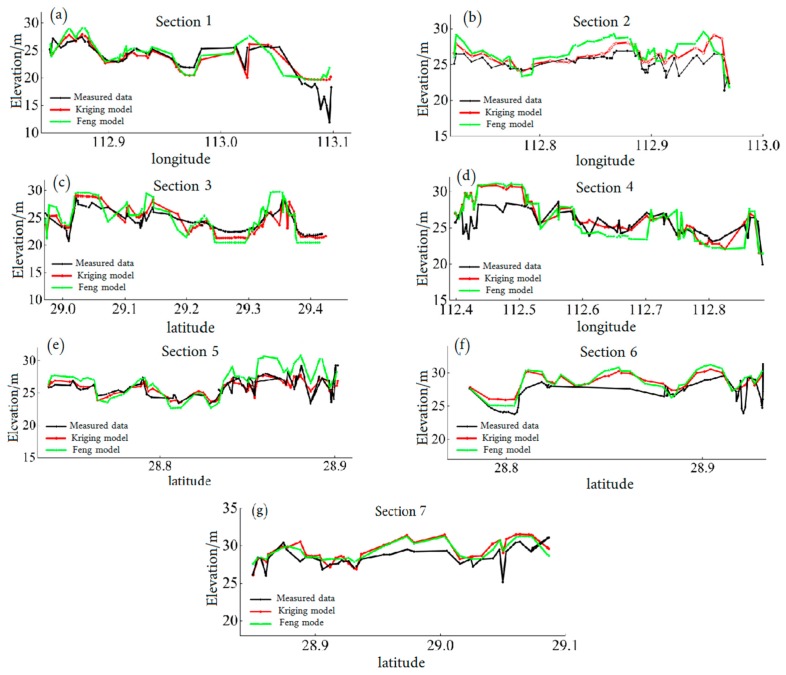
Comparison of measured and extracted cross sections.

Figure 15 is a schematic view of the position of the section. Seven sections were selected to compare the difference between the inversion topography and the measured topography. The comparison results are shown in Figure 16. These figures show that the Feng method is not effective in topography inversion because of the large amount of error involved in the water level inversion. The correlation between the latitude/longitude and the water level is unreliable. Accurately describing the variable water level of a dynamic lake proved to be difficult, and the water surface trend was misjudged. With the additional water level control points provided by the ICESat/GLAS data, the kriging method can perform water level inversion through the variogram without topographical information, and the final topography inversion error was small.

At the same time, the application of unconventional methods mentioned by Feng in the inversion of the bathymetry of Poyang Lake is limited to the bathymetry of the lake exposed to the air during the year. However, as Figure 16 shows, the bathymetry of the lake in the deep-water area, except for the area of Xiangjiang flood road, can also be obtained through unconventional methods although it is not exposed during the year. This proved that this method is not limited to the inversion of the bare lake bathymetry above the minimum water level. Most inland lakes have a relatively flat bottom. This method can be used to achieve the inversion of most bathymetry in the lake area.

According to the analysis of the results, a coarse-accuracy of bathymetry acquisition (error ± 1 m) can be achieved based on the method employed in this study. Although the accuracy has a gap when compared with the measurement accuracy of the advanced sounding technique, the data used in the present study comes from multiple remotely sensed images obtained free of charge, which can be used to extract the bathymetry of the lakes around the world. Even in remote areas where performing sounding is difficult, topography extraction can also be achieved with a short post-processing period. Thus, low-cost and rapid acquisition of coarse-accuracy bathymetry can be realized for lakes worldwide. If coarse-accuracy can meet the needs when sounding work cannot be implemented in a study area, or when measured topographical data proves difficult to obtain, the method employed here provides a good choice for obtaining the bathymetry of lakes.

### 5.2. Necessity of ICESat/GLAS Data

**Figure 17:**
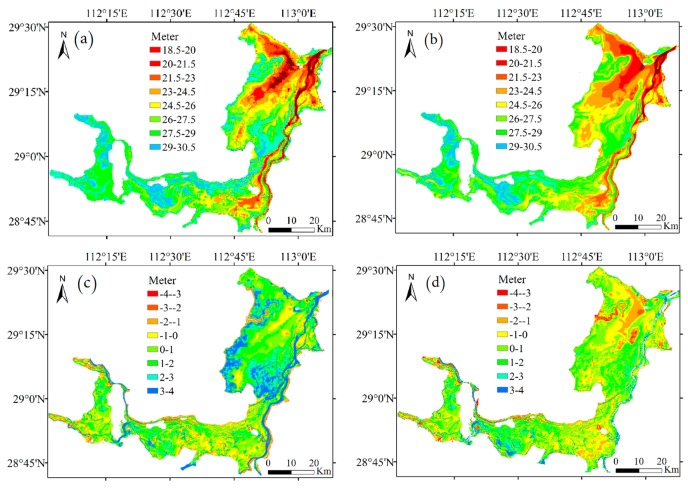
Comparison of the accuracy of topographic inversion before and after adding the ICESat/GLAS data: (**a**) before adding the ICESat/GLAS data; (**b**) after adding the ICESat/GLAS data; (**c**) distribution of the difference between the topographic inversion before adding the ICESat/GLAS data and measured elevation grid models; (**d**) distribution of the difference between the topographic inversion after adding the ICESat/GLAS data and measured elevation grid models.

The key to successful topographic inversion by unconventional methods is water level inversion. In the study of Poyang Lake, Feng used the measured water level in the lake area to perform water level inversion. In addition to using measured data at hydrological stations in the lake area, the present paper also introduced ICESat/GLAS data. Through the process mentioned above, the number of water level control points can be effectively increased. Figure 17 shows a comparison of the accuracy of topographic inversion before and after adding the ICESat/GLAS data. Figure 17 shows the addition of ICESat/GLAS has a significant effect on improving the accuracy of topographic inversion.

### 5.3. Innovation of the Bathymetry Inversion

In the present study, the unconventional method of inverting the bathymetry of a lake was studied in more detail to improve the accuracy. Two main aspects affecting the accuracy of this unconventional method include: accurate extraction of the lake boundary and of the water level inversion. For the exploration of the unconventional methods for large lakes, Feng used MODIS imagery in the study of Poyang Lake to perform the inversion of bathymetry of the lake based on the measured water level data from a hydrological station. In the present study, through stripe removal processing, the lake boundary was extracted from the Landsat series imagery with 30 m resolution. With a significantly better spatial resolution, more detailed and accurate bathymetry information has been obtained.

In the present study, kriging interpolation was utilized to invert the water level of the entire lake area based on the known water level control points. Despite of the complicated hydraulic conditions of Dongting Lake, the change of water level has been successfully reflected in the data. The experiment proves that kriging interpolation works better in water level inversion than the linear regression method proposed by Feng for water level inversion for a large lake without topographical data.

To improve the accuracy of water level inversion, this paper innovatively introduced the ICESat/GLAS data to increase the number of water level control points and maintain very accurate water level inversion in areas not covered by hydrological stations. Most researchers use ICESat/GLAS to test lake surfaces [39,40,41], but few researchers have employed ICESat/GLAS data to invert the bathymetry of a lake. In the present study, by combining the inverted strip-like topography with the lake boundary, the water level at this intersection was extracted as an additional water level control point. Experiments show that ICESat/GLAS data can better invert the striped topography. At the same time, the accuracy of topographical inversion can be effectively improved with the additional water level control points.

Because real data of bathymetry was lacking for other lakes, previous studies on unconventional methods can only be judged indirectly by the trend of erosion and siltation in the topography year by year. The accuracy of the inversion method could not be systematically analyzed while an analysis of uncertainty was also lacking. After completing the topography inversion, the present study used the measured topography to conduct a comprehensive accuracy analysis of the inversion results for the first time. The purpose was to study the causes of the errors and the reliability of the method.

### 5.4. Factors Affecting the Inversion Accuracy and Future Directions

**Figure 18:**
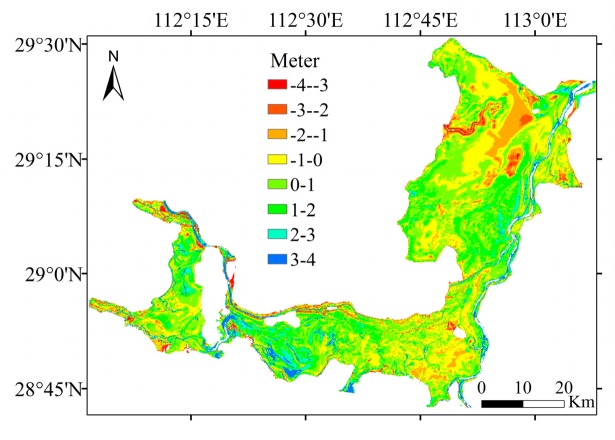
Distribution of the difference in the water level between the kriging and measured elevation grid models.

Although the kriging model has higher precision, this advantage is relative. Figure 18 shows the distribution of the difference in the water level between the kriging and measured elevation grid models. The error of the inversion results was mostly within ±1 m (Figure 18). There are a few abnormal points with an error greater than 3 m in topography with complex changes. Preliminary guesses of the cause of these errors are as follows. (1) The number of water level control stations is limited, and these stations cannot effectively cover the entire lake area. In some areas where the water level changes greatly with complex hydrodynamic conditions and no water level control station available, the water level inversion in these areas can generate large errors. (2) The low extraction accuracy of the lake boundary leads to the phenomenon that the boundary at different water levels overlaps in areas of steep topography or the high-water level boundary may intersect with the low water level boundary. Thus, a relatively poor inversion effect resulted for areas with complex and steep topography.

The key points of this method are the accuracy of both lake-boundary extraction and water level inversion. The spatial and temporal resolution of the lake area boundary can be improved by using multi-source high-precision remote sensing imagery. However, there is still much room for improvement in boundary extraction and water level inversion, and further research is needed.

**Figure 19:**
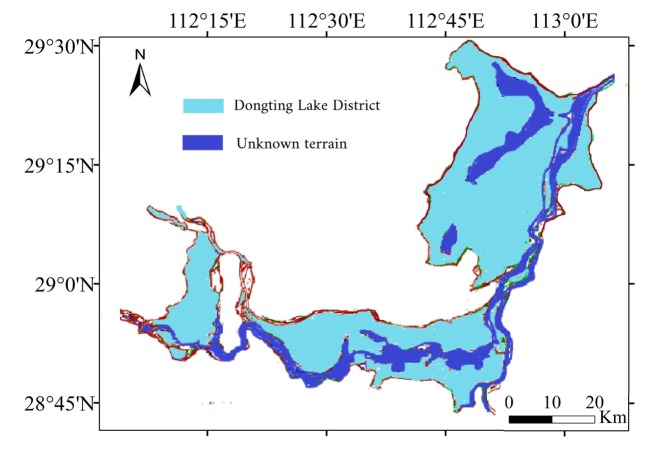
Ratio figure of unknown terrain.

At the same time, it should be noted that the shortcoming of this method is that it cannot obtain the topography of the water area below the lowest water level. Feng pointed out that the optical remote sensing image cannot capture the bathymetry of a point without exposure to the air. Although the surface area of Dongting Lake below the lowest water level only accounts for 15% (Figure 19), and most of the lake bottom topography can be obtained using this method, the acquisition of lake bottom topography below the lowest water level is still the focus of future research.

### 5.5. Advantages of the Proposed Method and Long Series of Observations

The unconventional method employed in the present study has advantages that are lacking for various sounding techniques such as single- or multi-beam sonar sounding and unmanned ship sounding. The conventional sounding techniques feature high measurement accuracy, but most have a long survey cycle with many difficulties in conducting surveys. The optical remote sensing inversion method makes up for this well, but the accuracy of underwater topographic surveys is not high, especially when applied to turbid water. Unconventional methods can rapidly acquire lake underwater topographical data using remote sensing combined with water level data and ICESat/GLAS data in a short cycle. Multi-phase remote sensing imagery obtained free of charge and frequent water level dynamic changes provided us with low cost but relatively accurate lake underwater topographical data.

The construction of both hydrodynamic and lake dynamic numerical models requires the corresponding bathymetry of the lake. Most of these topographical data are obtained from costly large-scale surveys made many years ago. The method employed in the present study can allow the bathymetry of a lake to be obtained rapidly, providing the boundary conditions of the numerical simulation for lakes that are impossible to survey or lack of measured data. Combined with water level data, the bathymetry can also be used to calculate the capacity of a lake to hold water resources, to establish a water level-lake capacity curve that will help researchers to understand changes in lake water resources [42,43,44,45], and provide important information for lake water assessment.

In addition, we proposed a new method to extract the water level based on ICESat/GLAS data in this paper. By extracting the strip-like topography of the lake area, the water level of the intersection is obtained by combining it with the water boundary on the day of data acquisition. The method can be applied to lakes that lack water level observation data acquired over a long time period and provide a new idea for studying the water level and water quantity change of lakes in remote areas.

With the development of GIS technology, GIS data can be combined with lake topography to evaluate flooding in a lake, such as understanding the extent of lake flooding and the estimation of the amount of floodwater remaining in a lake area. In addition, mastering our understanding of the bathymetry of lakes is of great significance to the shipping industry. Based on the difference between the current water level and the bathymetry of the lake, the water depth can be obtained to help ships avoid becoming grounded. In summary, obtaining coarsely accurate bathymetry mapping of a lake with an error of about 1 m based on the method employed in the present study has great significance for lake research and management evaluation in various fields.

## 6. Conclusions

Bathymetry can be indirectly reflected by the dynamics of lake water levels during a year. By taking Dongting Lake as an example, we constructed a bathymetry inversion model of Dongting Lake by combining remote sensing imagery, water level data, and ICESat/GLAS data. The accuracy of the model was evaluated by the measured underwater topography of the lake. The following main conclusions were obtained by comparing the measured data.

First, the underwater topography of Dongting Lake can be effectively obtained with relatively high accuracy through integrating water level data and multi-temporal Landsat TM/ETM+/OLI and ICESat/GLAS data. Furthermore, the inversion accuracy of kriging interpolation was found to provide better results than those of the Feng method. In addition, when the water level sample points cover the entire water area, the water level inversion accuracy is high, as the topographic inversion error can be reduced to ± 1 m. Third, the accuracy of kriging interpolation is not only related to the distribution of water level sample points, but also is related to the accuracy of the lake boundary extracted by remote sensing image. In waters with complex topography and steep slopes on the lake bottom, the boundary extraction accuracy was limited and large errors can be generated. The prediction accuracy of the waters with moderate slope changes was significantly higher than that of waters with complex topography. Finally, the addition of ICESat/GLAS data leads to more accurate water level inversion, which in turn greatly improves the topographical inversion accuracy. In addition, when underwater topographical data are not available, this study proved that kriging interpolation can be used to perform water level inversion by combining it with water level control points. Finally, the study shows that ICESat/GLAS data processing method is suitable for large lakes for obtaining water level control points, and accurate water level inversion can be achieved, thus greatly improving the accuracy of topography inversion.

## Figures and Tables

**Table 1:** Date of the remote sensing image and the sensor.

Path/Row	Acquisition Data	Sensor	Spatial Resolution (m)
123/40	2001/4/9	TM	30
123/40	2001/7/30	TM	30
123/40	2001/9/16	TM	30
123/40	2001/12/21	TM	30
123/40	2002/9/3	TM	30
123/40	2004/4/1	TM	30
123/40	2004/7/22	TM	30
123/40	2004/9/24	TM	30
123/40	2004/12/13	TM	30
123/40	2001/3/8	TM	30
124/40	2001/9/23	TM	30
124/40	2002/10/12	TM	30
124/40	2003/3/29	ETM	30
124/40	2003/8/28	TM	30
124/40	2003/10/7	ETM	30
124/40	2004/1/27	ETM	30
124/40	2004/3/15	ETM	30
124/40	2004/11/2	TM	30
124/40	2004/11/18	TM	30
124/40	2004/12/12	ETM	30

**Table 2:** Kriging interpolation with different semi-variogram models.

Kriging Method	Variogram	Mean	Root-Mean-Square	Mean Standardized	Root-Mean-Square Standardized	Average Standard Error
Ordinary Kriging	Circular	0.047	0.418	0.042	0.624	0.632
	Spherical	0.045	0.411	0.039	0.589	0.655
	Pentaspherical	0.046	0.407	0.041	0.552	0.684
	Exponential	0.023	0.412	0.022	0.479	0.798
	Gaussian	0.073	0.429	0.133	2.406	0.227
	Rational Quadratic	0.036	0.399	0.027	0.594	0.564
Universal Kriging	Circular	0.086	0.556	0.097	1.192	0.394
	Spherical	0.079	0.549	0.079	1.108	0.412
	Pentaspherical	0.072	0.548	0.064	1.054	0.431
	Exponential	0.061	0.555	0.048	1.006	0.474
	Gaussian	0.070	0.551	0.071	1.180	0.413
	Rational Quadratic	0.086	0.541	0.084	0.983	0.439
